# Isolated thalamic stroke – analysis of clinical characteristics and asymmetry of lesion distribution in a retrospective cohort study

**DOI:** 10.1186/s42466-021-00148-7

**Published:** 2021-09-13

**Authors:** Martin A. Schaller-Paule, Ariane Martinez Oeckel, Jan-Rüdiger Schüre, Fee Keil, Elke Hattingen, Christian Foerch, Maximilian Rauch

**Affiliations:** 1 Department of Neurology, University Hospital Frankfurt, Goethe-University, Schleusenweg 2 – 16, D-60528 Frankfurt am Main, Germany; 2 Brain Imaging Center, Goethe-University, Frankfurt am Main, Germany; 3 Institute of Neuroradiology, University Hospital Frankfurt, Goethe-University, Frankfurt am Main, Germany

**Keywords:** Diaschisis, Selection bias, Thalamus, Aphasia, Lateralization, Neuroradiology

## Abstract

**Background:**

More patients with left-hemispheric than right-hemispheric strokes are admitted to hospitals. This is due to the easier recognition of cortical symptoms of the dominant-hemisphere. The thalamus constitutes a “micro-model” of the brain cortex with structure-function relationships known to be asymmetric, especially for language, memory, and visuo-spatial neurocognitive functions. The goal of this study was to characterize clinical symptoms and lesion distribution patterns of patients with acute isolated thalamic stroke (ITS) and to evaluate whether left-sided lesions are overrepresented in the hospital.

**Methods:**

We performed a radiological database search including all brain scans performed in the Center of Neurology and Neurosurgery of the University Hospital Frankfurt between 2010 and 2019. A total of 5733 patients presenting with acute ischemic stroke were screened for ITS. Based on the MRI data, a lesion-overlap map was then generated to visualize the ITS lesion distribution.

**Results:**

Fifty-eight patients with unilateral ITS were identified. A majority of 38 patients (65.5%) showed left-sided ITS, whereas only 20 patients (34.5%) had right-sided ITS (*p* = 0.012). A particular difference was found for ITS lesions in the anterior thalamus of the anterolateral (*n* = 10) and anteromedian (*n* = 3) vascular territory, which were located in the left thalamus in 85% of patients (*p* = 0.011). No distribution difference was found for ITS lesions in the inferomedial (*n* = 7), central (*n* = 8), inferolateral (*n* = 23) and posterior (n = 7) vascular territories. The neuropsychological symptoms of thalamic aphasia (*n* = 8), neurocognitive impairment (*n* = 6), behavioral changes (*n* = 2), neglect (n = 2) and memory deficits (*n* = 3) were described predominantly in patients with left-sided ITS (*p* < 0.01). In contrast, other stroke symptoms (e.g., sensorimotor hemi-syndromes) did not reveal a side preponderance.

**Conclusions:**

The better recognizability of left anterior compared to right anterior thalamic stroke symptoms may have an impact on the frequency in which ITS patients are admitted to the hospital. Clinical characteristics of right anterior thalamic stroke should therefore be further investigated, and diagnostic instruments towards their detection be identified.

## Background

Left hemispheric strokes are overrepresented in hospitals when compared to right hemispheric strokes [1, 2]. This is attributed to a selection bias rather than a “true” numeric difference in stroke occurrence. Left hemispheric symptoms such as aphasia, alexia or apraxia can be recognized more easily by patients and next of kin than symptoms associated with the right hemisphere (such as neglect, visuo-spatial deficits or anosognosia). Thalamic strokes present with a wide variety of symptoms depending on their location, volume, and lateralization [3–5]. Numerous studies have already focused on syndromes based on the affected thalamic nuclei and vascular territories. However, it remained undetermined whether the distribution patterns of thalamic strokes admitted to a hospital also reflect left-right differences and are as such a “micro-model” of cortical stroke.

Four vascular territories are commonly defined for isolated thalamic stroke (ITS) [3, 4]. The (1) anterolateral territory is supplied by the tuberothalamic artery from the posterior communicating artery. Paramedian arteries arising from the pre-communicating segment (P1-segment) of the posterior cerebral artery (PCA) supply the (2) inferomedial territory, sporadically originating from an unpaired artery serving both sides [6]. Arising from the post-communicating P2-Segment of PCA, the thalamogeniculate arteries supply the (3) inferolateral territory and the posterior choroidal artery the (4) posterior territory (Table 1) [5, 7]. However, due to frequent variations in vascular supply and individual levels of collateralization the attribution of ITS to specific artery occlusion patterns has proven difficult [5, 8, 9].

**Table 1:** Vascular territories of the thalamus

Vascular territory and supplying artery	Affected Nuclei*	Clinical symptoms [4, 5, 8, 9, 11, 12, 22, 23]
**Anterolateral territory** from tuberothalamic artery arising from posterior communicating artery	Anterior thalamic nuclei group (AM, AV, AD) ventral amygdalo-fugal pathway ventral IML intralaminar nuclei (CL, CM, Pf) VA, RN rostral VL, ventral pole of MD mamillothalamic tract	Neuropsychological deficits: Decreased arousal, disorientation, mood disorders, personality changes, apathy, executive and drive impairment, perseverative behavior if left-sided: Thalamic aphasia, verbal short-term and visual memory impairment, amnesic syndrome, acalculia if right-sided: Hemi-spatial neglect, visuo-spatial processing deficits, executive cognitive tasks, non-verbal information processing memory impairment, amnestic syndrome
**Anteromedian territory** [22] varying arterial supply	MD intralaminar nuclei (CL, CM, Pf) IML	Hemiparesis, sensory loss, hemiataxia Neuropsychological deficits, memory impairment with severe anterograde amnesia, aphasia, agnosia and neglect, apraxia, executive dysfunction
**Inferomedial territory** from paramedian artery arising from pre-communicating P1-Segment (PCA)	MD posteromedial VL IML intralaminar nuclei (CL, CM, Pf) ventromedial pulvinar and LD mesencephalon (riMLF)	Acute: Sudden disturbance of vigilance; coma Chronic: “Thalamic dementia” with cognitive deficits, decreased arousal, impaired learning, memory deficits, apraxia, temporal disorientation, altered personality with apathy, mutism and lethargy, aggression, and agitation if left-sided: Thalamic aphasia if right-sided: Visuospatial processing deficits Motor and/or sensory hemi-syndrome, oculomotor nerve palsy, vertical gaze palsy
**Central territory** [22] varying arterial supply	VPL intralaminar nuclei (CL, CM, Pf)	Sensory loss, hemiparesis, ataxia Decreased vigilance, anterograde amnesia, short-term memory impairment
**Inferolateral territory** from thalamogeniculate artery arising from P2-Segment (PCA)	Ventroposterior complex (VPM, VPL, and VPI) VL MGB Pulvinar, LD	Sensory loss of all modalities, hemiparesis (often transient†), hemiataxia, predominantly on the right: Postlesion thalamic pain syndrome (Déjérine-Roussy-Syndrome) Hearing impairment Behavioral changes
**Lateral and medial posterior territory** from lateral and medial branches of the posterior choroidal artery arising from P2-segment (PCA)	Lateral: LGB, LD, LP, inferolateral parts of pulvinar, VPL, VL Medial: MGB, posterior parts of CM and CL, pulvinar	Dystonic posture, ataxic hemiparesis and sensory loss, visual field loss (hemi- or quadrant-anopia), neuropsychological features Sensory loss, hemiparesis, aphasia, memory impairment, hand tremor

* vascular supply varies between patients and anatomic conditions, the nuclei principally found in infarction in the given area are stated

† in inferolateral infarction the hemiparesis may be transient due to an edema with affection of the internal capsule

Abbreviations**:** anteromedial thalamic nucleus (AM), anteroventral thalamic nucleus (AV), anterodorsal thalamic nucleus (AD), intralaminar nuclei of thalamus (INT), ventral anterior thalamic nucleus (VA), ventral lateral thalamic nucleus (VL), medial dorsal thalamic nucleus (MD), internal medullary lamina of thalamus (IML), central lateral thalamic nucleus (CL), centromedian thalamic nucleus (CM), parafascicular thalamic nucleus (Pf), lateral dorsal thalamic nucleus (LD), ventral posterolateral thalamic nucleus (VPL), ventral posteromedial thalamic nucleus (VPM), ventral posterior inferior thalamic nucleus (VPI), lateral posterior thalamic nucleus (LP), medial geniculate thalamic nucleus/body (MGB), lateral geniculate thalamic nucleus/body (LGB), thalamic reticular nucleus (RN), fasciculus longitudinalis medialis (riMLF)

Structure-function relationships in the thalamus are generally asymmetric, an issue already raised in earlier studies on left-right lateralization based on electrical thalamic stimulation at time of stereotaxic operations [4, 10, 11]. It was shown for right handed patients that language processing, memory and neurocognitive functions are commonly mediated by the left anterior thalamus supplied by both or either tuberothalamic and paramedian arteries, while the right anterior thalamus serves as a relay in the prominent processing of visuo-spatial abilities and executive cognitive tasks (e.g. solving a complex maze), spatial awareness (hemi-spatial neglect), face-matching, and other non-verbal information processing [10, 12–15]. Consistently, no differences in lateralization were reported for sensory-motor functions (including thalamic pain syndrome), ataxia or hemianopia, as well as tremor and hemichorea, all commonly associated with inferolateral and posterolateral territory [4, 7, 16–18]. Unpaired vascular supply may lead to bilateral paramedian thalamic infarction, which frequently leads to acute vigilance impairment [19].

In summary, a subgroup of thalamic stroke patients may be overlooked in the prehospital setting due to less recognizable symptoms, and hence does not receive due stroke treatment in time. The aim of this study was to analyze the clinical symptoms and left-right lateralization patterns in isolated thalamic stroke patients to identify and further characterize those potentially missed stroke patients.

## Methods

### Study population

**Fig. 1:**
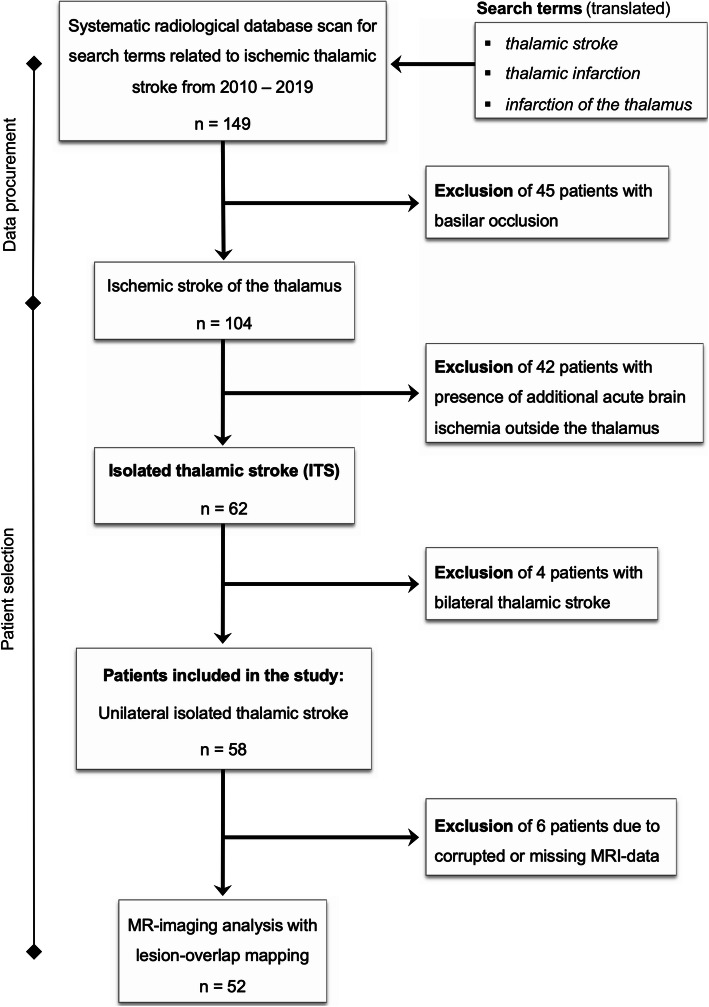
Study flowchart describing the data procurement and stepwise patient selection process intended for retrospective analysis

**Fig. 2:**
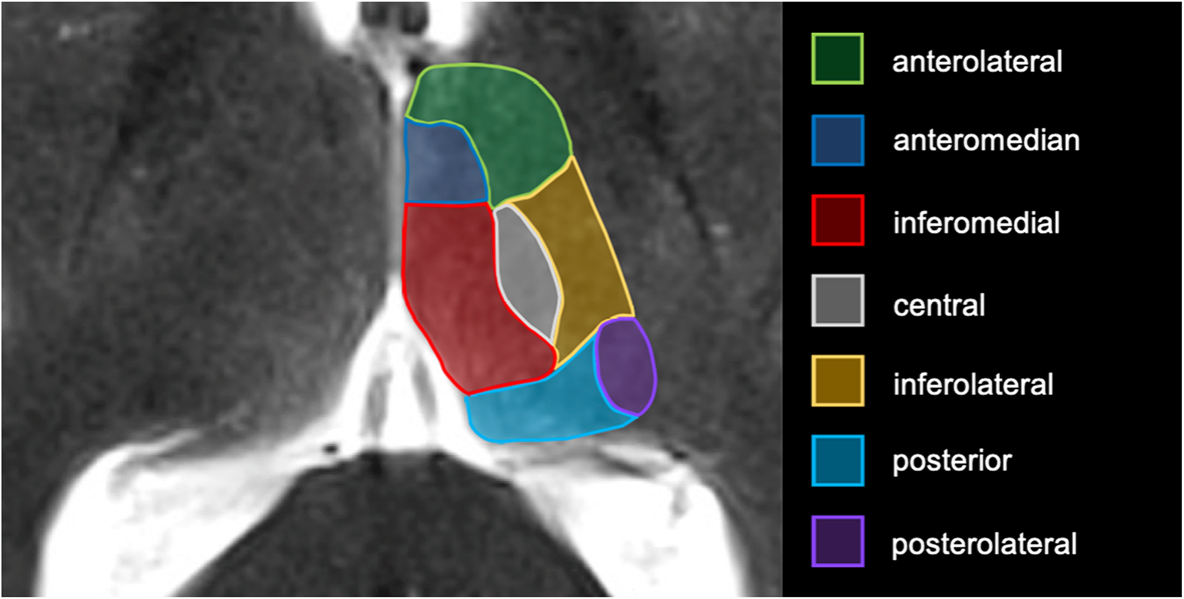
The thalamic vascular territories as defined in this study projected on an axial T2-weighted MRI template. The map was created based on the literature [4, 7, 9, 12, 22] and includes the four traditional and three variant type vascular territories

A systematic radiological database search for all thalamic strokes was undertaken for the years 2010 to 2019, scanning a total of 5733 patients presenting with ischemic stroke (ICD10 I 63) in the Center of Neurology and Neurosurgery of the University Hospital Frankfurt (Fig. 1). Cases with clinically and radiologically confirmed diagnosis of ITS only were included, and patients with concurring diagnoses were dismissed. We excluded 45 patients with underlying basilar occlusion and 42 patients with additional acute brain ischemia outside the thalamus (*non-isolated thalamic stroke*). We identified 62 patients with ITS, equaling 1.1% of all ischemic strokes. Four patients with bithalamic stroke were then excluded from further analysis. Clinical data was gathered and analyzed (M. S-P. and A. M. O.) for the remaining 58 patients with unilateral ITS. During stroke-unit treatment, patients did not undergo specific neuropsychological assessment tests, thus the neurocognitive information provided is based on the treating physicians’ clinical findings. In 57 of the patients, MRI data were available, one patient had CT only. First, interdisciplinary agreement on the dimensions of thalamic vascular territories was established based on current literature (Fig. 2). Individual lesion locations were then attributed to the vascular territories by two experienced neuroradiologists (M. R., F. K.) blinded towards the clinical data of the patients and the radiological report. In 29% (17/58), complete conformity was reached during blinded rating. Incongruent findings were then discussed with a third specialist in neuroradiology (E. H.) and jointly labeled in consensus. Ethical approval for the study was granted by the institutional Review Board of the Ethical Committee at the University Hospital Frankfurt (project-number: 20/616). All research methods were performed in accordance with the relevant guidelines and regulations.

### Lesion-overlap heat map

To address uncertainty caused by interrater variability, we performed a lesion-overlap study in MNI152 standard space to objectively investigate the distribution of thalamic stroke lesions on both sides. Diffusion-weighted (DWI) echo-planar image (EPI) data were aligned via three-dimensional T1-weighted data on the Montreal Neurological Institute MNI152 standard space template (1 mm isotropic resolution). For this purpose, T1-weighted data and DTI data were brain extracted and tissue segmented using the software tools BET and FAST from the FMRIB’S Software Library (FSL, version 5.0.7) toolbox. The DWI b = 0 data was aligned with the T1-weighted dataset via a boundary-based registration according to the segmented white matter. The T1-weighted data set was aligned to the MNI152 template using a combination of linear and non-linear registration. By combining the first (EPI to T1-weighted) and second (T1-weighted to MNI) transformation matrices, the transformation was then applied on the DWI b = 1000 dataset*.* In total, DWI data of 52 patients were transferred into the MNI152 standard space. Then, ITS infarct masks were manually marked based on DWI in MNI152 standard space and cumulated to generate a lesion heat map, which was projected onto the Oxford thalamic connectivity atlas [20]. Five corrupted MRI-datasets had to be dismissed in the process of data management.

### Statistical analysis

Patient demographics and baseline data were analysed by using summary descriptive statistics. Baseline differences between groups were tested by Welch’s two-sample t-test for continuous variables, Fisher’s exact test for categorial data, and the Mann–Whitney U test was used for non-normally distributed ordinal and continuous variables. Descriptive statistics were used to present baseline characteristics and results of outcome measurements. Differences between the occurrence of left- vs. right-sided lesions were evaluated by the exact binomial test. For all analyses, a level of significance of *p* < 0.05 was considered significant. R in version 3.6.1 was used for statistical calculations.

## Results

A total of 58 patients with ITS were included in the final analysis. Mean age was 62.8 years and 65.5% of patients were male. The majority of 38 patients showed isolated left-sided ITS (65.5%), while there were only 20 patients (34.5%) with ITS on the right side (*p* = 0.012). No significant differences between sides were found concerning stroke etiology and cardiovascular risk factors (Table 2). Patient admission within the 4.5-h time window (*p* = 0.16) and administration of thrombolysis (*p* = 0.18) did not differ significantly between sides.

**Table 2:** Baseline characteristics

Variable	All ITS *n* = 58	Left ITS *n* = 38	Right ITS *n* = 20
male sex, No. (%)	38 (65.5%)	25 (65.8%)	13 (65.0%)
age, mean (± SD)	62.8 (±15.6)	62.7 (±17.2)	62.9 (±12.6)
DWI lesion volume, median (IQR)	819 mm^3^ (531–1235)	923.5 mm^3^ (629–1361)	471 mm^3^ (268–876)
within time window < 4.5 h	21 (36.2%)	16 (42.1%)	5 (25.0%)
thrombolysis	7 (12.1%)	3 (7.9%)	4 (20.0%)
THALAMIC TERRITORY			
anterolateral	10 (17.2%)	8 (21.1%)	2 (10.0%)
anteromedian	3 (5.2%	3 (7.9%)	–
anterolateral + inferomedial	1 (1.7%)	1 (2.6%)	–
inferomedial	6 (10.3%)	4 (10.5%)	2 (10.0%)
central	8 (13.8%)	4 (10.5%)	4 (20.0%)
inferolateral	23 (39.7%)	12 (31.6%)	11 (55.0%)
posterolateral	7 (12.1%)	6 (15.8%)	1 (5.0%)
TOAST ETIOLOGY			
macroangiopathic	1 (1.7%)	1 (2.6%)	–
cardio-embolic	1 (1.7%)	1 (2.6%)	–
microangiopathic	41 (70.7%)	26 (68.4%)	15 (75%)
other	2 (3.4%)	2 (5.3%)	–
undetermined/ESUS	13 (22.4%)	8 (21.1%)	5 (25.0%)
CARDIOVASCULAR RISK FACTORS			
hypertension	44 (75.9%)	29 (76.3%)	15 (75.0%)
diabetes	18 (31.0%)	11 (28.9%)	7 (35.0%)
hypercholesterolemia	24 (41.4%)	14 (36.8%)	10 (50%)
atrial fibrillation	5 (8.8%)	3 (7.9%)	2 (10.5%)
smoker	22 (37.9%)	14 (36.8%)	8 (40%)
microangiopathy on MRI	29 (50.0%)	18 (47.4%)	11 (55%)

Abbreviations**:** *ITS* isolated thalamic stroke, *DWI* diffusion weighted imaging, *No.* number, *SD* standard deviation, *IQR* interquartile range, *ESUS* embolic stroke of undetermined source

Anterolateral and anteromedian territory ITS was found in 11 patients on the left and 2 patients on the right (*p* = 0.011). No significant differences in lateralization were found for central, inferomedial, inferolateral and posterior territory ITS. Thalamic aphasia was described in 8 patients with ITS on the left, but in none of the patients with right-sided ITS (*p* < 0.01). Neuropsychological impairment (vigilance deficits, neurocognitive impairment, behavioral changes, memory deficits, neglect) were apparent in 13 patients with left-sided ITS and in two patients with right-sided ITS (*p* = 0.003). In contrast, no lateralization difference was apparent for sensorimotor functions as well as coordinative and visual symptoms (Table 3). Visuo-spatial deficits were described in none of the patients on either side.

**Table 3:** Clinical characteristics

Variable	All ITS n = 58	Left ITS n = 38	Right ITS n = 20
GCS on admission (IQR)	15 (15 - 15)	15 (15 - 15)	15 (15 - 15)
NIHSS on admission (IQR)	2 (1 - 5)	2 (1 - 4.25)	3 (1.25 - 5)
NIHSS improvement on SU (IQR) ^1^	1 (0 - 3)	1 (0 - 2)	1.5 (0.25 - 3.75)
mRS at discharge (IQR)	0 (0 - 2)	1 (0 - 2)	0 (0 - 1)
Neurological findings on admission			
vigilance deficits	4 (6.9%)	3	1
neurocognitive impairment	6 (10.3%)	5	1
behavioral changes	2 (3.4%)	2	–
thalamic aphasia	8 (13.8%)	8	–
memory deficits	3 (5.2%)	3	–
neglect	2 (3.4%)	2	–
visuospatial deficits	none	–	–
dysarthria	15 (25.9%)	9	6
dysphagia	2 (3.4%)	1	1
hemiparesis	27 (46.6%)	16	11
sensory loss	34 (58.6%)	20	14
ataxia	7 (12.1%)	6	1
vertigo	5 (8.6%)	2	3
central facial paresis	8 (13.8%)	4	4
hemianopia	none	–	–
oculomotor deficits	3 (5.3%)	3	–
thalamic pain syndrome	2 (3.5%)	1	1
movement disorder	none	–	–

Abbreviations: *IQR* = interquartile range, *SU* = stroke-unit.

^1^ NIHSS improvement on SU is defined as the difference between NIHSS at admission to the certified stroke-unit and NIHSS at discharge from the stroke-unit

### Lesion-overlap map

**Fig. 3:**
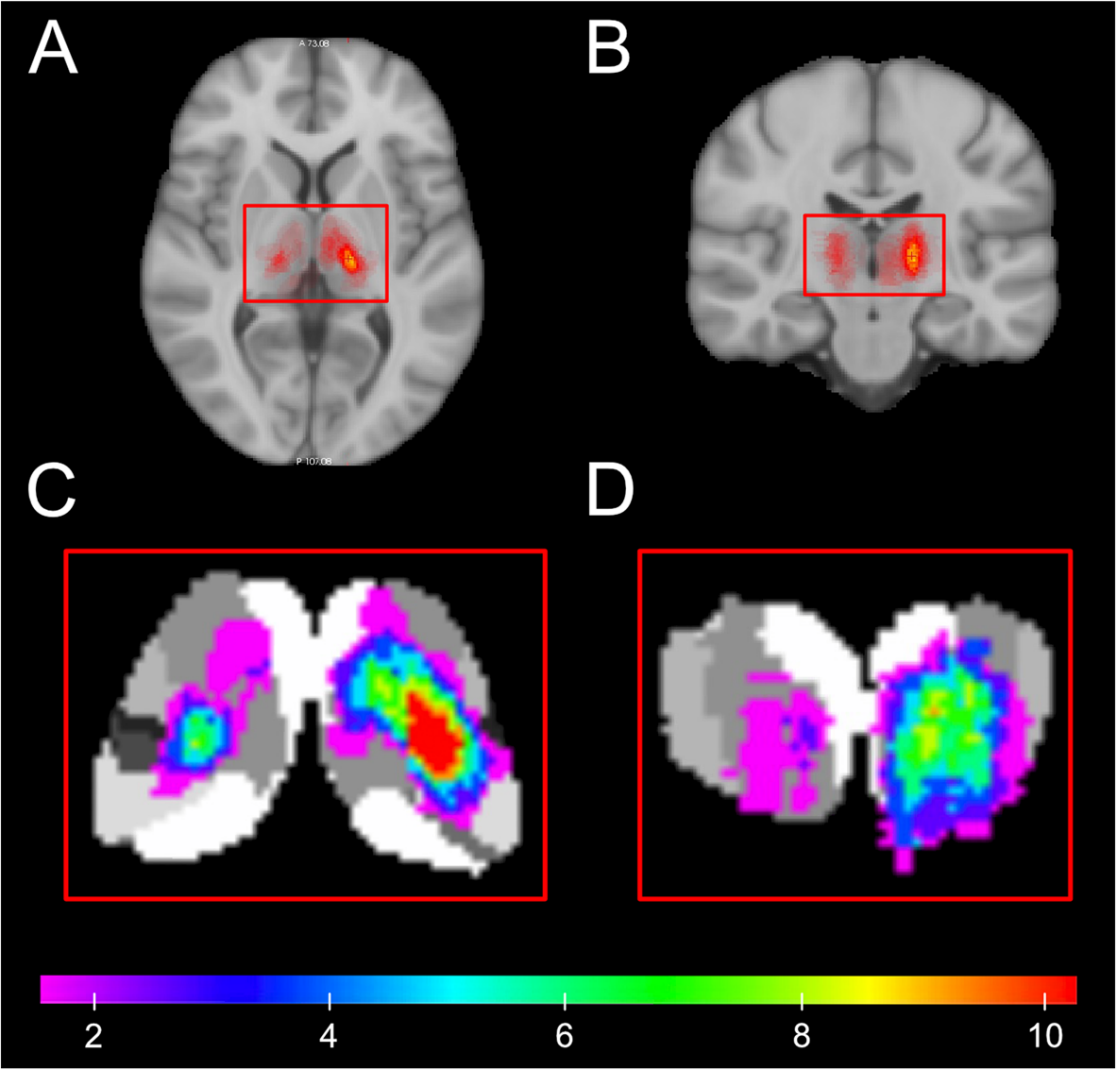
Lesion-overlap maps in axial and coronal plane of 52 thalamic stroke lesions. Upper row images illustrate the distribution patterns of thalamic strokes in axial (A) and coronal (B) slice in T1-weighted MNI standard space. A higher frequency of infarct lesions in the left thalamus is clearly visible. Lower row images provide a magnified lesion-overlap map of the thalamus in axial (C) and coronal (D) orientation, projected on seven sub-thalamic segments in standard space of the Oxford thalamic connectivity atlas [20]. The lesion distribution pattern as indicated by color visualizes the predominance of left anterior thalamic lesions in the anteromedian and anterolateral vascular territories when compared to right anterior thalamic lesions

A lesion-distribution map was generated using MRI data of 52 patients. As indicated by descriptive data, the left-over-right predominance of ITS can be visualized (Fig. 3). A lesion-overlap heat map focusing on the thalamus clearly illustrates the uneven distribution of strokes in the anterior thalamus with a preponderance on the left. In contrast, the other thalamic territories show a similar stroke frequency, as indicated by color. Based on the individual infarct masks, ITS lesion volumes were analyzed and showed significant larger infarct lesions on the left (median 924 mm^3^, 95% CI 879–1407 mm^3^) when compared to right-sided ITS-lesions (median 471 mm^3^, 95% CI 337–1096 mm^3^; *p* < 0.001).

## Discussion

The baseline data of our study cohort were in line with prior clinical studies on ITS, and the analyses met the assumed frequency of ITS patients (62 of 5733 ischemic stroke patients, equaling 1.1%) [13, 21]. To our knowledge, this is the first study focusing on the distribution pattern and lateralization of thalamic stroke lesions. If lateralization was specified in prior clinical studies, data frequently revealed a preponderance of left-sided lesions already. However, this was always left undiscussed, as authors chose to rather focus on the thalamic vascular territories [3, 11–13, 21–23]. Other clinical studies even withheld information on lesion lateralization and only differentiated unilateral and bilateral infarction [24]. In the current study, we allocated the ITS lesions into the vascular territories, as previously described in literature, and excluded non-isolated as well as bilateral thalamic strokes [3–5, 21]. To improve accuracy, we additionally allowed for the description of three distinct variant types (central, anteromedian and posterolateral territory) of thalamic borderzone ischemia [9, 22]. Noteworthy, we encountered a high interrater variability in the process, as vascular supply varies widely between patients, and ITS lesions seldomly fall only into one vascular territory. However, most ITS lesions could ultimately be allocated to one vascular territory in consensus.

This study demonstrated that patients with left-sided thalamic strokes were admitted to the hospital 1.9 times more frequently than patients with right-sided thalamic stroke. The finding correlated with a higher number of patients with ITS lesions in the left anterior thalamus. This asymmetry in anterolateral or anteromedian thalamic vascular territories could also be visualized on the lesion map (Fig. 2). Accordingly, review of clinical symptoms revealed a lateralization pattern for the neuropsychological symptoms thalamic aphasia, memory deficits, neglect, behavioral changes, and neurocognitive impairment, which were in 95% (20 of 21 patients) associated with left-sided ITS lesions. In contrast, no significant differences were found for lacunar syndromes (e.g., sensorimotor hemi-syndrome), similar to earlier findings for cortical stroke [1]. Therefore, an asymmetry of ITS lesions in the anterior parts of the thalamus most likely drove the predominance of left-sided ITS, and differences in the clinical symptoms may have largely contributed to this phenomenon. Thus, it can be hypothesized that an evenly large group of patients with right anterior ITS exists but was never admitted to the hospital.

To better understand the distribution pattern of this study, contributing factors and potential biases have to be evaluated. The real prevalence of right-sided ITS might be unequally lower in the population, consequently reflecting in the hospital admissions. However, this hypothesis is unlikely, since ischemic strokes were evenly divided in different population-based studies such as the Rotterdam study, and recent large MRI cohorts of lacunar stroke patients showed no side preponderance [2, 25]. In addition, there is no pathophysiological evidence for an hemodynamic asymmetry of ischemic events between left and right thalamus, as anatomical variations of the posterior circle of Willis such as fetal-type origin of PCA disperse equally [21]. Moreover, the search terms for thalamic stroke were deliberately defined by the study team. But although different search terms could have provided varying numbers of ITS patients, it is implausible that the chosen terms selected one side over the other.

Lastly, the chosen methodology of this study – a retrospective radiological database analysis – could have falsely preselected a specific patient group that is more likely to be admitted to a stroke-unit and receive brain imaging. For example, patients with aphasia may have been selected over patients with other, more subtle symptoms, because the latter might have been considered well treatable in the outpatient sector in advance. Also, primary care providers could have misinterpreted atypical stroke symptoms (e.g., subtle neurocognitive deficits) and attributed the complaints to another non-stroke condition. In addition, right-sided ITS patients themselves might not have presented to the healthcare system at all, because anosognosia is a common symptom of right thalamic lesions [26].

In summary, different clinical factors may have considerable impact on whether patients with right anterior ITS get admitted to a stroke-unit. For clarification, the clinical syndrome of right anterior ITS needs to be characterized and distinguished from left-sided ITS symptoms.

In the literature on thalamic functional anatomy, the anterior nucleus of thalamus (ANT) group was described as a key structure for emotional states, executive control, spatial navigation and episodic and visual memory function [27]. In analogy to cortical stroke, a ‘lateralized linguistic thalamus’ seems evident and recent research has also indicated ample support for a ‘lateralized neurocognitive thalamus’ [12, 15]. Univocally, thalamic (transcortical) aphasia and other, higher function language deficits such as retrieval of verbal short-term memory are commonly described in left thalamic lesions [10, 13, 28, 29]. Further neuropsychological symptoms associated with predominantly left thalamic lesions are constructional apraxia, agnosia, acalculia, behavioral alterations as well as dense amnesic syndrome, especially if the mamillothalamic tract is affected [11]. In contrast, research has indicated a right thalamic predominance for visual neglect, anosognosia, visual memory disturbances, simple speeded processing, mood regulation (depression, euphory, mania) and executive functions [9, 11, 23, 26]. However, literature is still inconsistent in the allocation of symptoms depending on lateralization, sometimes even contradictory [4, 11, 15]. Remarkably, the two patients in this study with right anterior thalamic stroke both showed only a lacunar (motor and sensorimotor) hemi-syndrome and dysarthria with a rapid recovery.

In this study, the lesion volume of right-sided ITS was smaller compared to left-sided ITS. This might be explained by the higher number of anterior ITS lesions on the left, that often spread wider and more extensive into the anterolateral and anteromedian territories. In contrast, right-sided ITS lesions consisted largely (75%) of small, strategic defects in sensorimotor pathways of the smaller central and inferolateral vascular territories, while only 42% of left-sided ITS patients had lesions in these locations (Table 2).

The thalamic stroke is a clinical chameleon with a wide range of symptoms far beyond the speech and sensorimotor systems. The clinical presentations of ITS in this study demonstrated that also sudden onset of a neuropsychological deficit is an indicator of thalamic stroke and should be valued accordingly. Though the clinical symptoms of right anterior thalamic stroke remain undetermined, review of literature suggests a syndrome consisting of subtle deficits in visuo-spatial navigation, visual memory impairment, memory function or emotional aspects. Widely used prehospital stroke screening tools such as FAST (Face, Arms, Speech: Time to call Emergency Medical Services) or NIHSS were created to be time-effective and therefore lack the ability to reliably detect neurocognitive deficits. Thus, patients with right-sided anterior thalamic stroke might not be identified and not receive optimized medical care within due time [30]. Even though less apparent neurological symptoms may not greatly affect patients’ everyday life, early detection of “silent strokes” is a crucial prerequisite for the timely initiation of secondary prevention in a significantly ageing population of stroke patients [31]. Examination techniques aimed to identify patients with right anterior ITS should therefore be tested in the prehospital sector, especially by primary care providers such as general practitioners and emergency personnel. A decrease of visuo-spatial ability or a hemi-spatial neglect could for example be handily assessed by mental screening tests such as Montréal Cognitive Assessment (MOCA) or Mini-Mental-State-Examination (MMSE). Both are widely available and could be used, if the onset of a neurocognitive deficit is acute or subacute, and help select patients for brain imaging. Since the neuropsychological symptoms of right anterior ITS may be hard to grasp, a low-threshold consultation of the in-house neuropsychologist may also be advisable. Future research should investigate examination techniques to identify right anterior ITS patients and measure their mental performances in neuropsychological test systems.

The number of potentially overlooked patients with right-sided ITS can be extrapolated. The German nationwide administrative database reported 227,542 acute ischemic strokes for the year 2017 alone [32]. With an assumed 1.1% rate of ITS among all reported strokes (*n* = 2503) and the inequality observed in this study of 65.5% left-sided and 34.5% right-sided ITS, approximately *n* = 777 right ITS patients may be overlooked every year in Germany.

Primary limitation of this study is the small sample size, that bears considerable risk of a sampling error. Though we identified no systematic methodological errors leading to the observed difference between sides, due to the small number of ITS patients, also minor biases in the patient selection process may have been impactful. However, this clinical study comprised 58 patients within a 10-year recruitment period in a tertiary care center and is amongst the largest clinical ITS patient samples in literature. Since ITS is not a common disease, recruiting considerable higher patient numbers for more reliable statistics is a major technical and organizational obstacle. Therefore, future studies assessing the ITS frequency should chose a multicenter design or use data from a state-wide registry. Furthermore, future population-based MR-studies with analysis of thalamic stroke patterns can provide more definitive insight into lesion distribution patterns. Noteworthy, a systematic neuropsychological testing did not take place in this study and we were dependent on information provided by the stroke physicians. For example, although visuo-spatial deficits or movement disorders were not described in this study, they may have stayed undetected due to a lack of neuropsychological testing. Noteworthy, a cumulative overlay of all larger lesions in the center of the thalamus on both sides should be considered when interpreting the lesion-overlap map.

## Conclusions

In summary, more recognizable symptoms of left anterior compared to right anterior ITS may have an impact on the frequency in which thalamic stroke patients are admitted to the hospital. In an unknown number of patients with right anterior ITS, the diagnosis of stroke might be overlooked by available screening methods, hindering patients from receiving medical treatment and secondary prophylaxis in time. This study demands for research aimed at characterizing clinical features of right anterior thalamus infarction, and identifying clinical instruments towards their diagnosis.

## Data Availability

Data and materials used in this study are available from the corresponding author upon reasonable request.

## Abbreviations

ANT: anterior nucleus of thalamus
EPI: echo-planar image
ITS: isolated thalamic stroke
MNI: Montreal National Institute
MRS: modified Rankin scale
NIHSS: National Institutes of Health Stroke Scale
PCA: posterior cerebral artery
